# Evaluation and Ranking for Scientific, Transparent and Applicable of Chinese Geriatrics Guidelines and Consensus Published in 2023

**DOI:** 10.1002/agm2.70100

**Published:** 2026-08-12

**Authors:** Daiping Li, Chao Gao, Xiaoli Huang, Ling Kang, Birong Dong, Jirong Yue, the Specialized Committee for Geriatric Medicine of the STAR, Daiping Li, Chao Gao, Xiaoli Huang, Ling Kang, Birong Dong, Jirong Yue

**Affiliations:** ^1^ Department of Geriatrics and National Clinical Research Center for Geriatrics West China Hospital of Sichuan University Chengdu Sichuan China; ^2^ Department of Editorial, Beijing Hospital, National Center of Gerontology, Institute of Geriatric Medicine Chinese Academy of Medical Sciences Beijing China; ^3^ Department of Geriatrics Chinese Academy of Medical Sciences, Peking Union Medical College Hospital Beijing China

**Keywords:** clinical practice, consensus, evaluation, geriatrics, guidelines

## Abstract

**Objectives:**

This study aims to evaluate the Scientific, Transparent, and Applicable Rankings (STAR) of guidelines and consensus published in Chinese geriatric medicine journals in 2023.

**Methods:**

The working group conducted a systematic search in eight major Chinese and English databases for guidelines and consensuses published by Chinese scholars in the field of geriatric medicine in 2023. The selected guidelines and consensuses were rated using the STAR rating tool, and the rating results were analyzed.

**Results:**

A total of 10 guidelines and 20 expert consensus documents were included. The STAR scores ranged from 9.4 to 55.7 with a mean of 29.3 ± 11.2. The STAR scores of 10 guidelines ranged from 16.1 and 55.7 with an average of 35.5 ± 13.1. The STAR scores of 20 consensus ranged from 9.4 and 48.5 with an average of 26.2 ± 8.9.

**Conclusion:**

Guidelines and consensus in geriatric medicine have shown significant improvements in 2023 compared to 2022 across registration, consensus methodology, working groups, and accessibility. However, areas such as protocol development, funding, and clinical issues require further refinement by the authors.

## Graphical abstract

Evaluation of geriatric guidelines and consensus in 2023 using the STAR tool shows improved scientificity and transparency compared to 2022, though protocol development, funding, and clinical issues need refinement. Collaborative efforts among developers, journals, and experts are key to further elevating quality.

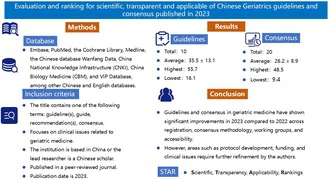

Clinical practice guidelines (referred to as “guidelines”) and consensuses provide important evidence for healthcare professionals to make clinical decisions [1]. High‐quality guidelines and consensuses can standardize medical practices, enhance healthcare quality, and reduce medical costs [2, 3, 4]. Scientific and standardized clinical practice guidelines and consensuses are crucial tools for guiding the diagnosis, treatment, health management, and comprehensive assessment of geriatric diseases, playing a significant role in improving healthcare quality and optimizing resource allocation. In recent years, the number of guidelines and consensuses in the field of geriatric medicine in China has grown substantially. However, variations still exist in their development methodologies, reporting quality, and clinical applicability. To ensure that these academic findings can truly serve clinical practice and policy formulation, it is particularly necessary to conduct a systematic evaluation of their scientific, transparent, and applicability.

In 2025, the World Health Organization Collaborating Centre for Guideline Implementation and Knowledge Translation, the Guidelines and Standards Research Center of the Chinese Medical Association Journal, and the Evidence‐Based Evaluation and Guideline Research Innovation Unit of the Chinese Academy of Medical Sciences jointly established the Scientific, Transparent, and Applicable Rankings (STAR) working group to evaluate guidelines and consensuses published in journals during 2023 [5]. The STAR working group comprises 40 specialty committees, involving 354 institutions. Among these, the Geriatric Medicine Specialty Committee expert group of the STAR working group is responsible for rating guidelines and consensuses in the field of geriatric medicine published in Chinese journals in 2023. In 2024, the Geriatric Medicine Specialty Committee of the STAR working group released the rating report for guidelines published by Chinese scholars in 2022, which generated a positive impact in the field of geriatric medicine [6]. This study aims to report the rating results of Chinese guidelines and consensuses published in journals during 2023, summarize identified issues, and continuously enhance the quality of guidelines and consensuses in the field of geriatric medicine.

## 1 Materials and Methods

### 1.1 Literature Search Strategy

We searched all publications from database from 1 January 2023 to 31 December 2023. The scope of database searches included: PubMed, Web of Science, Scopus, SinoMed, Chinese Medical Journal Full‐text Database, Wanfang med online, China National Knowledge Infrastructure (CNKI) and Wanfang Data. Searches were conducted using subject headings and free‐text terms. Chinese and English search terms included: guideline, recommendation, consensus.

### 1.2 Inclusion and Exclusion Criteria and Literature Screening

**Inclusion criteria:**

1. The title contains one of the following terms: guideline(s), guide, recommendation(s), consensus.
2. Focuses on clinical issues related to geriatric medicine.
3. The institution is based in China or the lead researcher is a Chinese scholar.
4. Published in a peer‐reviewed journal.
5. Publication date is 2023.

**Exclusion criteria:**

1. Duplicate publication that has already undergone STAR rating.
2. Journal republication of documents released by government agencies on their official websites.
3. Interpretations or reviews of guidelines or consensus.
4. The development working group consists of fewer than 10 members.
5. When contacting the corresponding author via email to request supporting materials for the guideline or consensus, special circumstances such as confidentiality make the document unsuitable for rating.

Guidelines and consensuses in the field of geriatric medicine were selected based on the specialty of the lead development institution, the specialty of the corresponding author, and the specialty covered by the journal of publication.

### 1.3 Working Group and Rating Process

In September 2025, the STAR Geriatrics Specialty Committee organized 1 chairperson, 4 vice‐chairpersons, 20 committee members, and 8 young members to jointly conduct the rating of guidelines and consensuses in the field of geriatric medicine. Before the formal evaluation, all raters participated in unified training and completed an examination on the STAR integrated rating platform. Those who passed the examination were qualified to participate in the rating work. All raters signed the STAR Working Group's Conflict of Interest Declaration Form via the integrated rating platform, and members with conflicts of interest were not allowed to participate in the rating of relevant guidelines or consensuses. During the formal evaluation, rating tasks were assigned by the chairperson, vice‐chairpersons, or secretary. Two raters from the Geriatrics Specialty Committee independently completed the rating based on the full text and supporting materials of the guideline or consensus using the STAR integrated rating platform and verified the rating results. Any disagreements were resolved through internal discussion or consultation with secretariat members. The rating results were reviewed by the chairperson, vice‐chairpersons, or secretary, ultimately producing the 2023 Rating Results for Guidelines and Consensus in China's Geriatric Medicine Field.

### 1.4 Statistical Analysis

Statistical analysis was performed using Microsoft Office Excel 2021 (version 16.60). The scoring summary of the included guidelines or consensuses was evaluated using item score ratios and domain score ratios.

$$\begin{array}{c} \begin{array}{cc} \text{Item score ratio}=\; & (\text{Total score of the item across}\;\mathrm{all}\;\text{guidelines or consensuses}\\ & /\text{Theoretical total score of the item})\times 100\%\\ \text{Domain score ratio}=\;\; & (\text{Total score of items within the domain across}\;\mathrm{all}\;\text{guidelines or consensuses}\\ & /\text{Theoretical total score of the domain})\times 100\% \end{array} \end{array}$$

## 2 Results

### 2.1 Selected Guidelines and Consensuses

**FIGURE 1:**
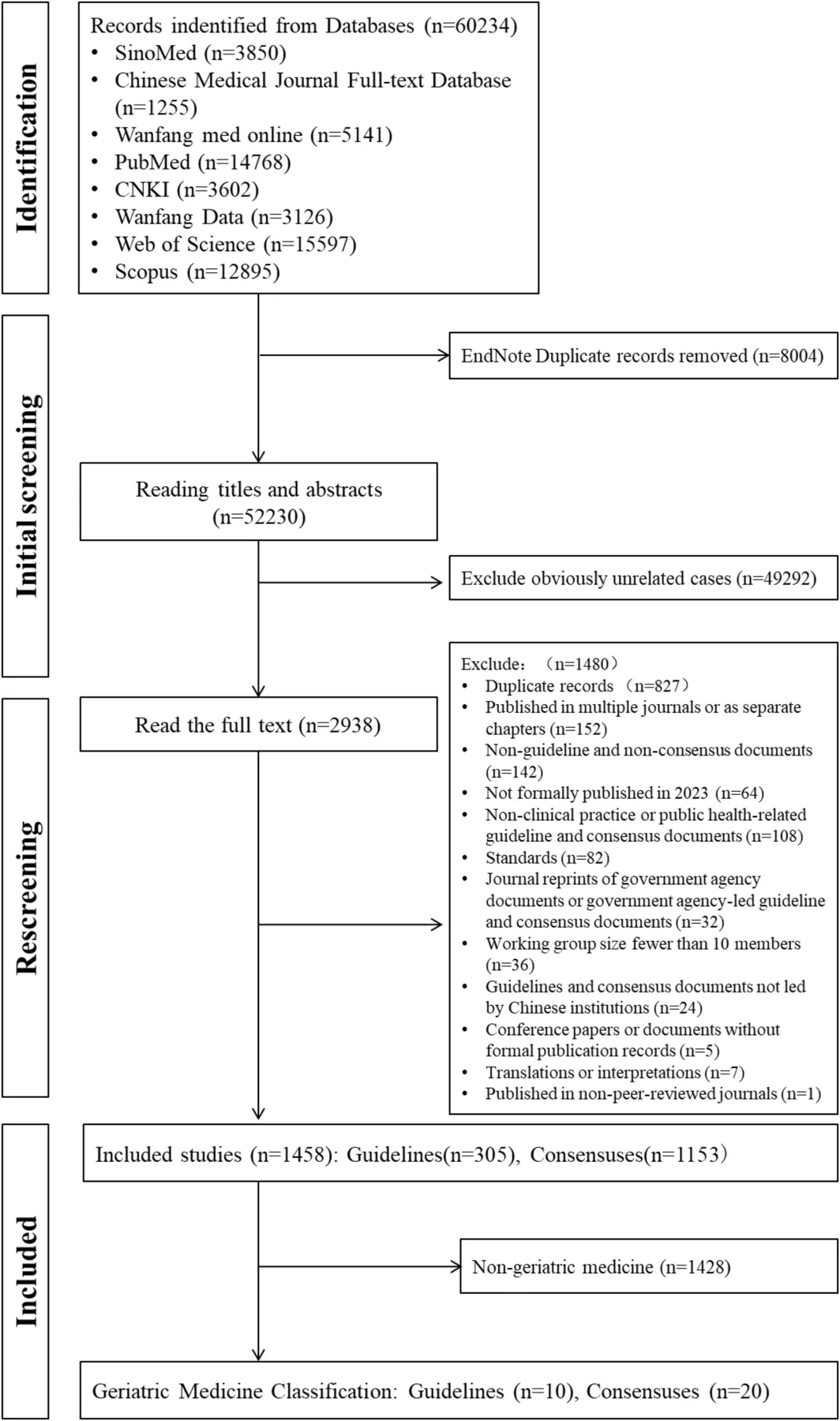
Literature screening flowchart.

Literature screening process is shown in Figure 1. After retrieval and screening, a total of 10 guidelines and 20 expert consensuses [7, 8, 9, 10, 11, 12, 13, 14, 15, 16, 17, 18, 19, 20, 21, 22, 23, 24, 25, 26, 27, 28, 29, 30, 31, 32, 33, 34, 35, 36] in the field of geriatric medicine were included, with the detailed list presented in Table 1. The guidelines primarily focused on the following areas: nutrition, cognition, frailty, type 2 diabetes, chronic obstructive pulmonary disease, osteoarthritis, disability, sleep, and digital memory in older adults. The consensuses primarily addressed research domains such as gastroesophageal reflux, venous thromboembolism, sarcopenia, COVID‐19 infection, cardiovascular diseases, frailty, diabetes, chronic musculoskeletal pain, multimorbidity, nutrition, sleep, resilience, and rational drug use in older adults. *Chinese Expert Consensus 2023 on the Prevention of Venous Thromboembolism in Critically Ill Older Patients* was published in two journals [10, 37], and *the Chinese expert consensus on prevention and control interventions for older adults with sarcopenia 2023* had both Chinese and English versions [17, 38]. Despite contacting the authors, no supporting materials regarding the development process of the guidelines or consensuses were provided. Consequently, the ratings were based solely on the published full‐text content.

**TABLE 1:** List of Chinese Geriatric Medicine Guidelines and consensus published in journals in 2023.

Number	Author	Title	Type
1	China Association of Gerontology and Geriatrics Cognitive Neuroscience and Health et al. [7]	Chinese clinical practice guidelines for digital memory clinic	Guidelines
2	Chinese Geriatrics Society et al. [8]	Practical guideline on nutritional therapy in critical geriatrics in China (2023)	Guidelines
3	Liu et al. [9]	Chinese expert consensus on diagnosis and management of gastroesophageal reflux disease in the elderly (2023)	Consensus
4	Critical Care Medicine Division of Chinese Geriatric Society et al. [10]	Expert consensus on the prevention of venous thromboembolism for elderly patients with critical illness in China (2023)	Consensus
5	Cognitive Disorders Branch of Chinese Geriatric Society et al. [11]	Guidelines for cognitive screening and follow‐up in Guangdong‐Hong Kong‐Macao Greater Bay Area (2023)	Guidelines
6	Geriatrics Branch of Chinese Medical Association et al. [12]	Clinical practice guideline on endocrine system aging and hormone management for elderly with frailty in China (2023)	Guidelines
7	Chinese Gerontological and Geriatric Society [13]	Guidelines for Chronic Disease Management of Osteoarthritis in the elderly	Guidelines
8	Wu et al. [14]	Chinese expert consensus on prevention and control interventions for older adults with physical functional impairment(2022)	Consensus
9	Chinese Gerontological and Geriatric Society [15]	Guidelines for the Management of Chronic Obstructive Pulmonary Disease in the Elderly	Guidelines
10	Chinese Gerontological and Geriatric Society [16]	Guidelines for Chronic Disease Management of Type 2 Diabetes in Older Adults	Guidelines
11	Cui et al. [17]	Chinese expert consensus on prevention and intervention for elderly with sarcopenia (2023)	Consensus
12	Qi et al. [18]	Expert consensus on diagnosis and treatment of COVID‐19 in the elderly in China (Grass‐roots doctors edition)	Consensus
13	Gerontology Working Group of the Cardiovascular Disease Branch, Chinese Medical Association et al. [19]	Chinese Expert Consensus on the Assessment and Management of Cardiovascular Disease with Frailty in Older Adults	Consensus
14	Zhang et al. [20]	Chinese expert consensus on antithrombotic management of high‐risk elderly patients with chronic coronary syndrome	Consensus
15	Shi et al. [21]	Chinese Expert Consensus on the Management of Comorbidities of Cardiovascular and Cerebrovascular Diseases and Diabetes in Older Adults	Consensus
16	Ji et al. [22]	Expert consensus on management of geriatric chronic musculoskeletal pain (2023)	Consensus
17	Zhu et al. [23]	Chinese expert consensus on management of elderly patients with multimorbidity (2023)	Consensus
18	Health Management Branch of the Chinese Geriatrics Society et al. [24]	Expert Consensus on the Clinical Application of Immunonutrients	Consensus
19	Zhejiang Medical Association Subcommittee on Cancer Nutrition and Therapy et al. [25]	Expert Consensus on Nutritional Diagnosis and Treatment for Elderly Patients	Consensus
20	Chinese Society of Gerontology and Geriatrics [26]	Guidelines for Chronic Disease Management of Coronary Heart Disease in the Elderly	Guidelines
21	He et al. [27]	Consensus on Dietary Guidance and Nutritional Supplement Use for Sarcopenia	Consensus
22	Beijing Dietetic Association et al. [28]	Chinese Expert Consensus on Perioperative Nutritional Assessment for Elderly Cardiac Patients	Consensus
23	Peripheral Vascular Disease Management Branch of the Chinese Geriatrics Society [29]	Chinese expert consensus on the prevention and treatment of venous thromboembolism in the elderly	Consensus
24	Clinical Nutrition Branch of the Chinese Nutrition Society et al. [30]	Expert Consensus on Nutritional Intervention for Elderly Patients with Sleep Disorders	Consensus
25	Ma et al. [31]	Chinese expert consensus on multidisciplinary decision models for the elderly with malnutrition (2023)	Consensus
26	Expert Panel for Resilience Assessment in Geriatric Infection Patients [32]	Expert Consensus on Resilience Assessment for Elderly Patients with Infections	Consensus
27	Chinese Society of Gerontology and Geriatrics [33]	Guidelines for the Management of Chronic Insomnia in Older Adults	Guidelines
28	Geriatric Endocrinology Committee Jiangsu Provincial Geriatric Medicine Association et al. [34]	Expert Consensus on the Application of SGLT‐2 Inhibitors in Elderly Patients with Type 2 Diabetes	Consensus
29	Shandong Geriatrics Society [35]	Guidelines for Prevention and Intervention of Disability and Semi Disability among the Elderly in Shandong Province	Guidelines
30	Wang et al. [36]	Consensus on the comprehensive management of evaluation and intervention of rational drug use in the elderly	Consensus

### 2.2 Evaluation Results

**FIGURE 2:**
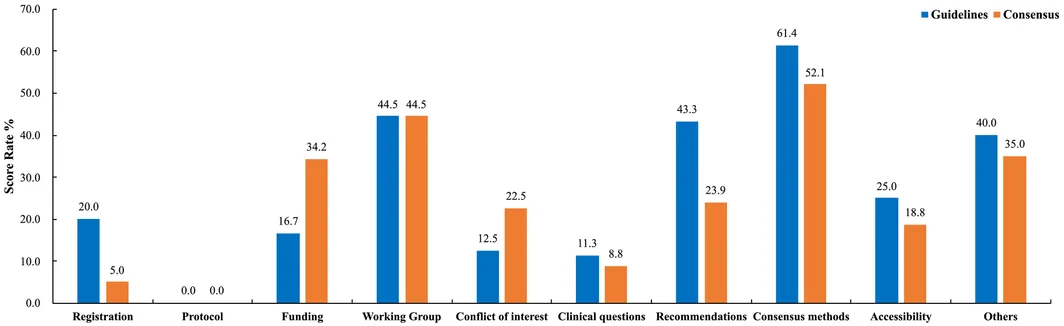
Domain score rates of Chinese Geriatric Medicine Guidelines and consensuses published in journals in 2023.

**FIGURE 3:**
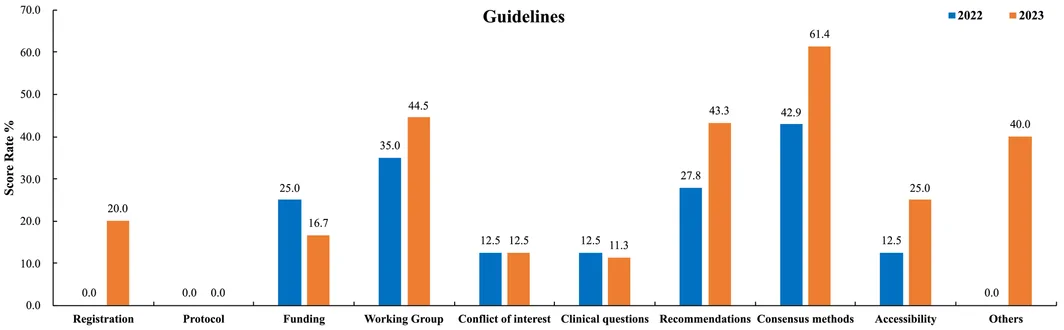
Domain score rates of Chinese Geriatric Medicine Guidelines published in journals in 2022 vs. 2023.

**FIGURE 4:**
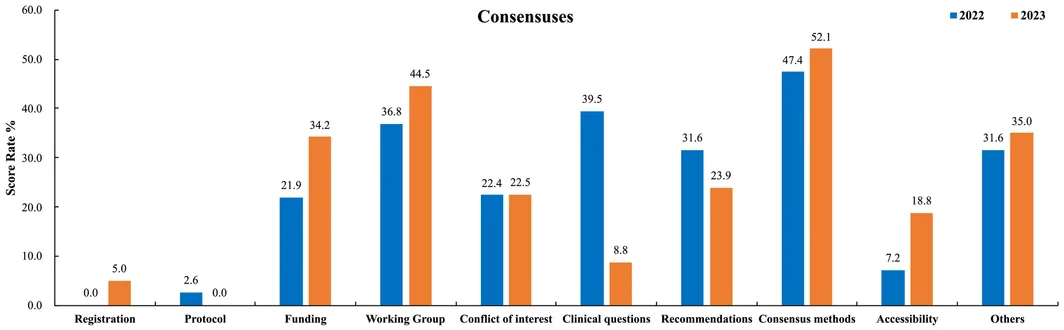
Domain score rates of Chinese Geriatric Medicine Consensuses published in journals in 2022 vs 2023. Domain score ratio = (Total score of items within the domain across all guidelines or consensuses/Theoretical total score of the domain) × 100%.

The STAR scores of the 30 guidelines and consensuses ranged from 9.4 to 55.7 with a mean of 29.3 ± 11.2. Among these, the STAR scores of 10 guidelines ranged from 16.1 and 55.7 with an average of 35.5 ± 13.1. The STAR scores of 20 consensus ranged from 9.4 and 48.5 with an average of 26.2 ± 8.9. The highest scoring domain for both guidelines and consensus documents was “Consensus Methodology” at 61.4% and 52.1%, respectively, while the lowest scoring domain for both was “Plan Description” at 0.0%. Detailed scoring rates are presented in Figure 2. The comparison of scoring rates between guidelines and consensuses published in 2022 and 2023 is shown in Figures 3 and 4. Scoring rates for specific items are detailed in Table 2.

**TABLE 2:** Item score rates of Chinese Geriatric Medicine Guidelines and Consensuses published in journals in 2023.

Domain	Item	Item score	Guidelines score (%)	Consensuses score (%)
Registration	1. Registered	1.5	20.0	5.0
	2. Provided platform and registration number for registration	3.5	20.0	5.0
Protocol	3. Wrote a protocol	1.9	0.0	0.0
	4. Protocol is publicly accessible (e.g., available on the registration platform or website)	3.1	0.0	0.0
Funding	5. Disclosed funding sources	1.0	30.0	70.0
	6. Described the role of funding in guideline development	0.9	0.0	0.0
	7. Stated that recommendations were not influenced by funding	1.3	20.0	32.5
Working group	8. Disclosed the institutions of participants	0.9	100.0*	100.0*
	9. Described the grouping of participants	1.0	60.0	42.5
	10. Specified the roles and responsibilities of participants	1.3	0.0	20.0
	11. Explicitly included members from two or more disciplines other than the core specialty	1.3	50.0	55.0
	12. Explicitly stated that the working group included methodologies or evidence‐based medicine experts	2.8	40.0	5.0
Conflict of interest	13. Disclosed conflicts of interest	4.4	25.0	37.5
	14. Provide detailed conflict of interest management procedures	4.8	0.0	7.5
Clinical questions	15. Clearly defined the clinical questions the guideline aimed to address	6.4	20.0	10.0
	16. Described the methods for collecting clinical questions (e.g., literature review of guidelines, systematic reviews, primary studies; user surveys; expert consultations)	2.5	20.0	25.0
	17. Described the process for selecting clinical questions	3.4	0.0	0.0
	18. Clinical questions were structured using the PICO (Population/Patient, Intervention, Comparison, Outcome)	4.8	5.0	0.0
Recommendations	19. The primary recommendations are supported by explicit references.	1.7	100.0*	100.0*
	20. Described systematic evidence retrieval methods	2.2	30.0	2.5
	21. Specified inclusion and exclusion criteria for evidence	1.5	20.0	0.0
	22. Assessed the risk of bias or methodological quality of the evidence	1.9	5.0	2.5
	23. Conducted synthesis and analysis of evidence results	2.1	30.0	7.5
	24. Described the criteria for grading the quality of evidence	2.2	90.0	7.5
	25. Provided evidence summary tables or grading criteria	2.4	5.0	0.0
	26. Traceable to full‐text of systematic review	1.7	60.0	15.0
	27. Listed clinical questions lacking evidence and suggested future research directions	1.2	50.0	80.0
Consensus methods	28. Described the consensus methods used for recommendations (e.g., Delphi method, nominal group technique, consensus conference, GRADE grid)	5.1	30.0	20.0
	29. Described how consensus was reached based on factors other than evidence quality (e.g., economics, patient preferences and values, benefit‐harm balance, accessibility, equity, acceptability)	3.8	50.0	60.0
	30. Provided detailed records of the consensus process	1.8	15.0	17.5
	31. Recommendations were clearly listed (e.g., using tables, enlarged or bolded text, underlining)	4.1	80.0	82.5
	32. Specified the strength of each recommendation	6.3	75.0	17.5
	33. Provided explanatory notes for each recommendation	3.9	80.0	72.5
	34. Described considerations for implementing the recommendations	2.8	100.0*	95.0
Accessibility	35. Guidelines are published through multiple platforms (e.g., guideline libraries, conferences, online platforms)	2.5	10.0	0.0
	36. Provide guides for different user versions	1.4	0.0	0.0
	37. Published guidelines or recommendations in additional formats (e.g., images, videos)	1.1	0.0	0.0
	38. Guides are available for free.	2.3	90.0	75.0
Others	39. Provided a recommended Pathway for the recommendations	1.2	40.0	35.0

* Represents the item with the highest score rate in the guidelines.

## 3 Discussion and Suggestions

The overall scores indicate that in 2023, the average scores of guidelines and consensuses published in Chinese geriatric medicine journals in 2023 were 35.5 and 26.2, respectively. Compared to the scores from 2022 (24.5 and 30.2), the overall score for guidelines increased by 44.9%, while the score for consensuses decreased by 13.2%. The quality of guidelines and consensuses improved to varying degrees across multiple domains, but the protocol domain remains inadequately addressed.

A comparative analysis of the specific rating outcomes for guidelines and consensuses reveals that the quality of Chinese geriatric guidelines in 2023 was significantly higher than that of consensuses. The score rates for multiple guideline domains exceeded the consensus, but the consensus significantly surpassed guideline scores in the areas of funding and conflicts of interest. Geriatric medicine guidelines demonstrated significant improvements compared to 2022 in the domains of registration, working groups, evidence, consensus methods, accessibility, and other areas. However, further enhancements are still needed in the domains of protocol development, funding, and clinical question formulation. Geriatric consensuses have shown significant improvement compared to 2022 in the domains of registration, funding, working groups, consensus methods, accessibility, and other areas. However, there remains room for quality enhancement in the domains of protocol development, clinical question formulation, and evidence.

The seven items with the lowest score rates were: 3 (Wrote a protocol), 4 (Protocol is publicly accessible), 6 (Described the role of funding in guideline development), 17 (Described the process for selecting clinical questions), 36 (Provide guides for different user versions), and 37 (Published guidelines or recommendations in additional formats). The above items have not yet received sufficient attention by guidelines and consensus developers. It is recommended to enhance the dissemination of methodological knowledge to promote quality improvement.

In terms of rating innovation, compared to the previous year, this year adopted the STAR integrated rating platform for unified management. This includes training and examinations for rating personnel, authors uploading supporting materials, managing conflicts of interest among rating personnel, assigning rating tasks, conducting intelligent pre‐evaluations through the platform, rating personnel performing evaluations, and reviewing rating outcomes. This further enhances the standardization of the rating process, as well as the transparency and scientific of the rating procedure.

### 3.1 Limitations of the Ratings

Firstly, no supporting rating materials were received for this year's evaluation; ratings were conducted solely based on the full texts of the guidelines and consensuses. Consequently, the rating results may not be comprehensive. Secondly, the STAR ratings primarily assess the scientific, transparent, and applicable nature of the guideline and consensus development processes. They do not evaluate the specific content of the recommendations, nor do they reflect the clinical significance of the recommendations in practice. Therefore, the rating results should be interpreted in conjunction with the clinical relevance of the recommendations to provide meaningful reference for healthcare professionals.

### 3.2 Recommendations

(1) Developers of geriatric guidelines and consensuses may refer to the 2022 [6] and the rating results released in this edition to enhance the standardization of their guideline and consensus development processes in a targeted manner. (2) It is recommended that guideline and consensus expert panels strictly adhere to the rating criteria to ensure quality control throughout the development process. (3) Journals are encouraged to require authors submitting guideline or consensus manuscripts to provide supporting materials documenting the development process. (4) Healthcare providers and patients may consider both the STAR rating results and the recommendations themselves to comprehensively select appropriate guidelines and consensuses. Through collaborative efforts across multiple stakeholders, the overall quality of geriatric medicine guidelines and consensus statements can be further elevated.

## Funding

This work was supported by Sichuan Province science and technology innovation base project (2023ZYD0173).

## Conflicts of Interest

The authors declare no conflicts of interest.

## Data Availability

The data that support the findings of this study are available from the corresponding author upon reasonable request.
